# Influence of Wheat-Milled Products and Their Additive Blends on Pasta Dough Rheological, Microstructure, and Product Quality Characteristics

**DOI:** 10.1155/2013/538070

**Published:** 2013-10-07

**Authors:** B. Dhiraj, P. Prabhasankar

**Affiliations:** ^1^Department of Biotechnology, Vellore Institute of Technology, Vellore, India; ^2^Flour Milling, Baking and Confectionery Technology Department, CSIR-Central Food Technological Research Institute, Mysore 570 020, India

## Abstract

This study is aimed to assess the suitability of *T. aestivum* wheat milled products and its combinations with *T. durum* semolina with additives such as ascorbic acid, vital gluten and HPMC (Hydroxypropyl methyl cellulose) for pasta processing quality characteristics such as pasta dough rheology, microstructure, cooking quality, and sensory evaluation. Rheological studies showed maximum dough stability in Comb1 (*T. aestivum* wheat flour and semolina). Colour and cooking quality of Comb2 (*T. durum* semolina and *T. aestivum* wheat flour) and Comb3 (*T. aestivum* wheat semolina and *T. durum* semolina) were comparable with control. Pasting results indicated that *T. aestivum* semolina gave the lowest onset gelatinization temperature (66.9°C) but the highest peak viscosity (1.053 BU). Starch release was maximum in Comb1 (53.45%) when compared with control (44.9%) as also proved by microstructure studies. Firmness was seen to be slightly high in Comb3 (2.430 N) when compared with control (2.304 N), and sensory evaluations were also in the acceptable range for the same. The present study concludes that Comb3 comprising 50% *T. durum* semolina and 50% *T. aestivum* refined wheat flour with additives would be optimal alternate for 100% *T. durum* semolina for production of financially viable pasta.

## 1. Introduction

Population growth has increased the demand for food; rising prosperity has increased the demand for quality food. At the same time, consumers demand convenience foods, since they are becoming increasingly health conscious; therefore, there is a need to diversify food products. Wheat is being used as a staple food for most part of the world, because of its special dough characteristic like cohesiveness and thus being used in the preparation of bread and other wide ranges of products like noodles, soups, pasta, and other foods like biscuits, cookies, cakes, and breakfast cereal [1].

Pastas include noodles in various lengths, widths and shapes and varieties that are filled with other ingredients like ravioli and tortellini. Pasta is an excellent source of complex carbohydrates, which provide a slow release of energy. Unlike simple sugars that offer a quick, yet fleeting boost of energy, pasta helps sustain energy. Pasta is very low in sodium and cholesterol-free. Per cup, enriched varieties provide a good source of several essential nutrients, including iron and several B-vitamins.

 In recent years, pasta has become more popular due to its nutritional properties [2]. Increase in popularity of pasta products and their increased consumption make it very important for increase in availability of raw materials. Durum wheat, being the hardest of wheat's is used at large scale for pasta production. Being commercially expensive to produce due to the limited availability makes it a case of study. The endosperm made up of Durum is completely different from other wheat species because the mineral was distributed throughout endosperm and has more carotenoids contents, low protein efficiency ratio, and excellent rheological characteristics which are desirable for making pasta products.

 However, over the years, numerous studies on alternate methods for production of pasta from different raw materials have been conducted, where alternates for raw materials were used for production focusing on reduced cost and to match similar parameters and to improve nutritional value. Durum wheat accounts for about 16% of the total wheat production. Unlike common wheat, there is only one predominant class of durum wheat. Limited availability of durum wheat is noticed due to the increase in consumption of pasta products, making it commercially expensive for procurement of raw material. On the other hand, pasta products are becoming quite popular all over the world. However, the availability of durum wheat for production of pasta products is very limited. 

Alternates are thus researched upon, where it is found that the common wheat widely available throughout the world is similar in comparison with durum wheat. The percentages of starch, protein, minerals, lipids, and amino acids are roughly equivalent. Common bread wheat occupying 90% of the world total production makes it a cheap and readily available alternate for pasta production, thus saving money and creating ample opportunities to improve nutritional quality.

The objective of the present study is to find suitability of *T. aestivum* milled products (refined wheat flour, semolina, and whole wheat flour) in the place of *Durum* semolina for preparation of pasta, thereby reducing the cost of production, maintaining or improving the quality of the product, and then study in detail the rheological properties of the pasta dough, chemical composition, nutritional profile, and quality of developed pasta products.

## 2. Materials and Methods

### 2.1. Raw Material

Commercially available semolina of *T. durum* was procured from the local market. Freshly prepared refined wheat flour, whole wheat flour, and semolina were obtained from Narasu's Roller Flour Mills, Salem, Tamil Nadu, India. All the flour samples were stored at room temperature until further use. All reagents and chemicals used are of analytical grade (AR) unless otherwise specified.

#### 2.1.1. Raw Material Characterization

Raw materials were analyzed for particle size (AACC 55-30.01), moisture content (AACC-44-15A), ash content (AACC-80-01), gluten content (AACC-38-10), and Micro-Kjeldahl method was used to determine nitrogen contents of pasta samples (AACC, 2000), sedimentation value (AACC-56-70), Farinograph (AACC-54-21), Mixograph (AACC 54-40.02), and Alveograph characteristics (AACC-54-30A) using the mentioned standard methodologies.

### 2.2. Pasta Preparation

Raw materials and water were premixed in a spar mixer at speed 1 (60 rpm) for 10 min to facilitate uniform distribution of water. The premixed dough (500 g) was transferred to a laboratory pasta machine (La Monferrina, Model Dolly, Asti, Italy). The dough was then extruded through the brass die for pasta type Shells in the required size and was dried in Sakar Drier (Shirsat, Mumbai) at 75°C for 4 h. The pasta samples were then allowed to cool at room temperature and then packed in polyethylene covers for storage. Similar method was also followed for the other wheat-refined products. Formulation for preparation of different pasta samples was given in Table 1.

### 2.3. Quality Characteristics of Pasta

#### 2.3.1. Cooking Quality

Cooking time for pasta samples was estimated according to AACC method 66-50 [3]. Cooking loss was determined according to the Bureau of Indian Standards (BIS 1976). Twenty-five grams of pasta sample was weighed and put in the 250 mL of boiling water. Start timer count is and stirs well to make sure that the pieces are separated. Check the piece of pasta after every 30 sec intervals for its hydration and cooking by squeezing the sample.  Stop cooking when the core portion just disappears. The gruel was drained and collected for quantification of solid leach out. Cooked pasta samples were analyzed for its texture, colour, and sensory evaluation.

#### 2.3.2. Pasta Firmness

Firmness of cooked pasta was measured according to method adopted by Krishnan and Prabhasankar [4] using a universal texture measuring system (LLOYDS Instruments, LR-5 K, Hampshire, UK).

#### 2.3.3. Sensory Characteristics

A panel consisting of 25 panelists (*n* = 25), who were regular eaters of pasta, was employed for the sensory evaluation of pasta samples. Product characterization was carried out under “daylight” illumination and in isolated booths [4]. Briefly, panelists evaluated the randomly coded pasta samples for their colour, appearance, aroma, texture, taste, and overall acceptability. Assessors were instructed to cleanse their palate with cold, filtered tap water before tasting each sample. The overall sensory attributes were measured using Hedonic scale of 1–9 where 9 = Like extremely, 8 = Like very much, 7 = Like moderately, 6 = Like slightly, 5 = Neither like nor dislike, 4 = Dislike slightly, 3 = Dislike moderately, 2 = Dislike very much, and 1 = Dislike extremely. All the parameters were carried out in quadruplicates and the means values were reported. 

#### 2.3.4. Colour Measurement

The values of surface colour (*L*, *a* and *b*) of raw pasta in terms of lightness (*L*) and colour (+*a*: red −*a*: green; +*b*: yellow; −*b*: blue) and Δ*E* were measured using Hunter Lab colour measuring system (Colour measuring LabScan XE system, USA). All the parameters were carried out in quadruplicates and the means values were reported.

### 2.4. Microstructure

The cooked pasta samples were freeze-dried using Heto freeze dryer (DW3, Allerod, Denmark). Surface and cross section of freeze-dried samples were mounted on the specimen holder and sputter-coated with gold (2 min, 2 mbar). Finally, each sample was transferred to the microscope where it was observed at 15 kV and a vacuum of 9.75 × 10^−5^ Torr. A scanning electron microscope (Leo 435 VP, Leo Electronic Systems, Cambridge, UK) was used to scan the images.

### 2.5. Dietary Fiber

The method was followed from AOAC [5] method 32.1.17.

### 2.6. *In Vitro* Starch Hydrolysis

*In vitro* digestibility of starch was analyzed using the method of Englyst et al. [6], with minor modification. Freeze dried and ground sample (50 mg) was dispersed in 4 mL of sodium acetate buffer (pH 4.6, 0.4 M) containing amyloglucosidase and was incubated in water bath for 30 min at 60°C. Then, the enzyme was inactivated by placing the tubes in boiling water bath (100°C) for 15 min. The tubes were cooled to room temperature and then centrifuged at 5000 rpm for 10 min. Supernatant was measured for its glucose content using a glucose oxidase-peroxidase (GOD-POD) kit (Autospan, Span Diagnostics limited, India). Absorption was measured at 505 nm, and the glucose concentration was converted into starch content using a 0.9 factor. Each sample was analyzed in triplicates. 

## 3. Results and Discussion

### 3.1. Raw Material Characterization

#### 3.1.1. Granulation

The particle size distributions of the flour samples were determined with a series of standard sieves, and the results were expressed as a percentage of the sample weight (Table 2). Ideally, the majority of semolina particles should fall within a narrow range of particle size range so that pasta dough water uptake will be homogenous. It was observed that the refined wheat flour (refined wheat flour) is much finer than the control (semolina). The nonuniformity in the particle size can be attributed to the grinding of the semolina particles, which was carried out by an external minimill grinder. However, the results obtained were good within the requirements for good pasta making quality. Whole wheat was ground to a granulation similar to that of the semolina (Table 2). Particle size affects the rate of hydration of the milled product during pasta processing [7]. Incomplete hydration of semolina or ground whole wheat would result in white specks in the spaghetti. White specks are starchy areas of little or no gluten development. Thus, white specks would affect the appearance, mechanical strength, and cooking quality of the spaghetti [8].

#### 3.1.2. Chemical Analysis of Raw Materials

Proximate analysis of all the raw materials (refined wheat flour, whole wheat flour, Semolina) used were shown in the Table 3. It is noticed that Comb1 (*T. aestivum* wheat flour & semolina with additives) with the presence of additives has moisture of 10.87% is very much higher compared to the control (9.8%). The other two blends (Comb2 & Comb3) had acceptable levels of moisture compared to the control as they have the presence of semolina. The proximate composition of all the samples was found to be within limits of PFA and ISI standards. Refined wheat flour was used in Comb1 which caused the increase in moisture content. Similar results of semolina were also reported by [9] where moisture of Indian durum varieties varied from 9.0 to 11.5%, ash content varied from 0.79 to 0.86%, and the protein content varied between 12.1 and 15.9%. Ash, an index of the mineral content of the flour, is of much relevance, and in that it gives the indication of the grade or the extraction of the flour. This is because of the low level of mineral content present in the endosperm when compared to the outer bran content. The bran layer is rich in ash and protein, so removing bran during milling would lower the protein and ash content. It is noticed that all the comb samples have lower ash content when compared to the control, where Comb3 (semolina (*T. aestivum*) and semolina (*T. durum*)) (Table 3) has 0.596%. Comb2 & Comb1 samples also have lower ash values, thus improving the quality of the flour. A significant increase in protein content is noticed with the comb samples (Table 2), which could be due to the addition of additives. This is similar to the results reported by Prabhasankar et al. [10] for increase in nutritional attributes of pasta samples with the addition of additives. The maximum increase is noticed in Comb2 (refined wheat flour and semolina (*T. durum*)) which is 15.40% (Table 3) when compared to the control which has 12.8% (Table 3) of protein. Increase is also noted in Comb1 at a level of 13.70%, thus imparting better nutritional value.

### 3.2. Rheological Characterization

#### 3.2.1. Mixograph

**Figure 1:**
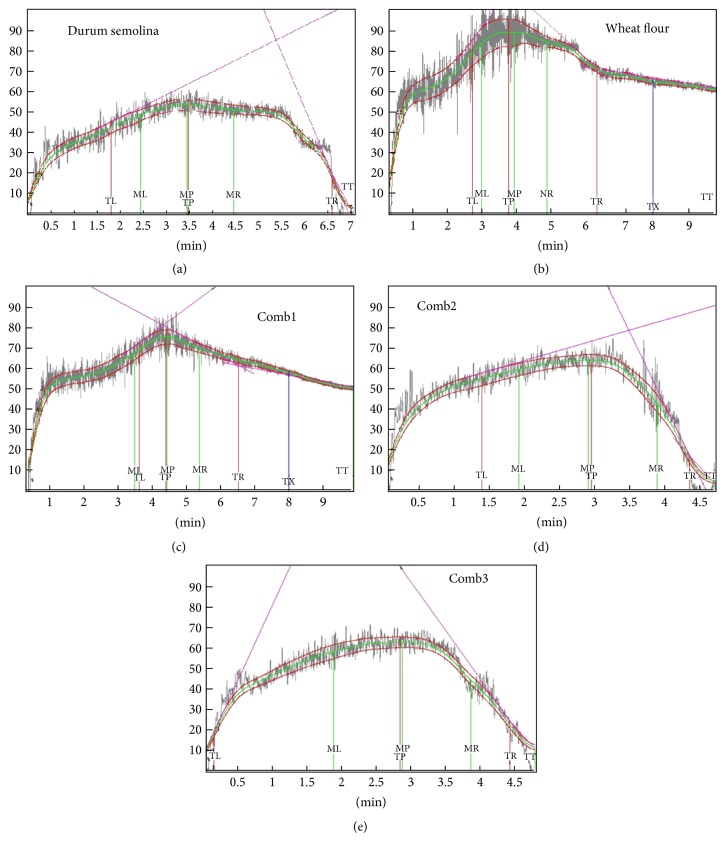
Mixograph characteristics of flour and blend samples. (a) Control (durum); (b) wheat flour; (c) Comb1; (d) Comb2; (e) Comb3.

The Mixograph is a primary physical dough testing procedure in the US durum wheat-breeding program [11].  Figure 1 details the results of the monograms obtained from the pasta samples. Comparing the two raw materials used, dough strength is greatly deferred between the samples where Figures 1(b) and 1(c) were comparatively stronger than the others. This is because of the presence of refined wheat flour, which is generally high in strength compared to semolina. Regardless of samples, refined wheat flour had higher peak heights as shown in Figures 1(b) and 1(c), but semolina had higher peak width as shown in Figures 1(a), 1(d), and 1(e). Higher peak proves that the flour is very resistant to extensibility and also has greater mixing ability making it more stable as seen in control (Figure 1(b)). But semolina generally is seen to have a lower peak height (Figure 1(a)), thereby reducing the extensibility of the flour making it better for pasta making.

Mixogram dough development time (the time required for the mixogram curve to reach maximum height) was greater for Comb1 had the highest (4.26) which was made up of refined wheat flour. Based on the mixograms obtained, refined wheat flour had higher dough stability when compared to semolina as evidenced by the rapid decline of the mixogram curves after 3.5 min. Dough stability indicates the time during which the dough resists mechanical action without undergoing a change in consistency [12]. Lack of dough stability or tolerance to over mixing could be related to the dilution of semolina with bran or germ as fewer storage proteins are available to form a gluten matrix. Other researchers have reported that dough stability decreases with increase in bran concentration [13]. In spite of all these issues, all the parameters were in accordance was the norms and were able to produce good pasta quality.

It is seen from (Figure 1(d)), that the addition of refined wheat flour along with semolina has given the flour additional properties showing a higher peak height when compared to control (Figure 1(a)), thus aiding in better pasta making ability.

#### 3.2.2. Farinograph

The results obtained along with the amount of water absorption required to centre the Farinogram curve on the 500 BU (Brabender Units) line varied as shown in Table 4. It is well noticed from the results that the samples with a higher concentration of refined wheat flour exhibit stronger dough characteristics (increased water absorption, dough development time and dough stability), that is, refined wheat flour & Comb1 in contrast to weak dough development. The whole wheat flour showed higher water absorption 63.9% due to high damages starch, whereas the dough development time and dough stability were lower than those in the whole-wheat flour. The similar kind observation was also noticed by Vetrimani et al. [14]. The addition of gluten as indicated in samples Combs 1, 2, and 3, increased water absorption, dough development time and dough stability was seen. It was also noticed that stability and dough development time are related with each other, and increase in development time caused increased stability, thus leading to stronger dough. Samples with maximum wheat bran concentration caused increase in water absorption with whole wheat flour (whole wheat flour) having maximum absorption (65.9%) (Table 4) which was also noticed by [15] and, lowest for refined wheat flour (Comb1). Rosell et al. [16] reported that the differences in water absorption are mainly caused by the greater number of hydroxyl group which exist in the fibre structure and allow more water interaction through hydrogen bonding.

#### 3.2.3. Alveograph

**Figure 2:**
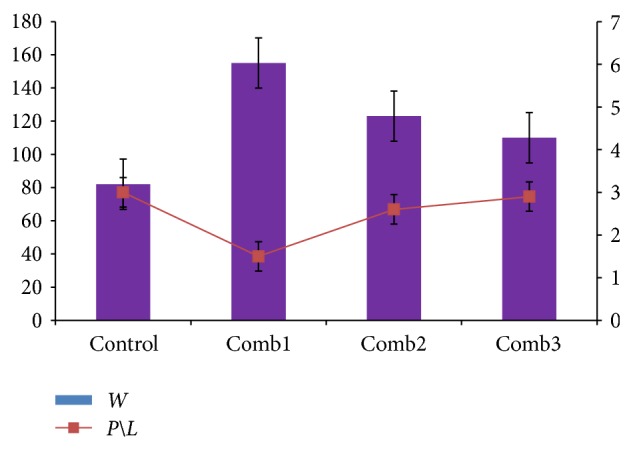
Comparison of Gluten strength value (*W*) to Curve configuration ratio (*P*∖*L*). Control (*T. durum *semolina), Comb1: *T. aestivum* wheat flour (50%) & semolina (50%) with additives, Comb2: refined wheat flour (50%) and *D. semolina* (50%) with additives, Comb3: *T. aestivum* semolina (50%) and *D. semolina* (50%) with additives.

Alveograph is used to measure the viscoelastic properties (strength and extensibility) of the gluten protein that correlate's well with the firmness and springiness of cooked pasta [17]. Gluten strength and tenacity/extensibility ratio *P*∖*L* is a good predictor of cooking quality, thus making it relevant for pasta-making ability.  Figure 2 results obtained from the Alveograph of the various samples of flour. Regarding the bread making and pasta characteristics of the flour under examination, the Alveograph indices (*W* and *P*∖*L*) values are considered. The most dramatic effect of additives addition on semolina was observed when the biaxial properties of the wheat dough were assessed in the Alveograph. The addition induced a significant modification of the Alveograph parameters, where a steady increase of the tenacity (*P*) besides to a significant decrease in dough extensibility (*L*). Overall effect on tenacity and extensibility led to a significant increase of the curve configuration ratio (*P*∖*L*). Similar results were obtained by Bonet et al. [18] where he noticed the same, with increase in GO (glucose oxidase) concentration. The same was also noticed in regard to GO by [19]. But the only change was seen in the deformation energy (*W*) where a steady decrease was noticed in this case. This could be attributed to the addition of a percent of refined wheat flour as the raw material. As the percent of refined wheat flour reduced, The *P*∖*L* ratio increased which was very similar to that of control. Thus leading to better pasta quality. Whereas Bonet et al. [18] noticed a steady increase as there was no presence of refined wheat flour and also the addition of GO cause additional protein cross-links resulting in larger *P*∖*L* values [20].

### 3.3. Analysis of Pasta

#### 3.3.1. Colour Measurement

Colour of pasta is a key quality because of the vital impact on the point of sale. In pasta products made with semolina, the higher the value, the more desirable the product [21]. Among *L*, *a*, and *b* parameters, the first two are considered more important as colour attributes. Hunter colour parameters (*L*, *a*, *b*) of raw samples of durum pasta (control) the other raw materials used along with the SA and the comb pasta are shown in the Table 5. The lightness values for 100% semolina pasta were in the range of 69–76. The higher values are noticed in Table 5, which shows increased levels of pasta quality and the presence of no adulteration in the pasta samples proving high levels of economical advantage as well as appearance. The lightness is seen lower in the all the samples, this may be attributed due to the alteration with different wheat milled products which has a lower lightness index compared to the control, thereby lower carotenoid pigments which contribute to the colour of durum pasta. Yellowness in all samples was seen lower than the control, and this is due to the same factors affecting the lightness index. It is noted that the Comb3 blend shows the highest lightness, but the Comb2 blend gives the highest yellowness when compared to the control. It is also noted that the influence of the additives did not cause any major difference in both indexes. But it is seen that the influence of additives has reduced the lightness but has increased the yellowness.

#### 3.3.2. Pasta Cooking Quality

**Figure 3:**
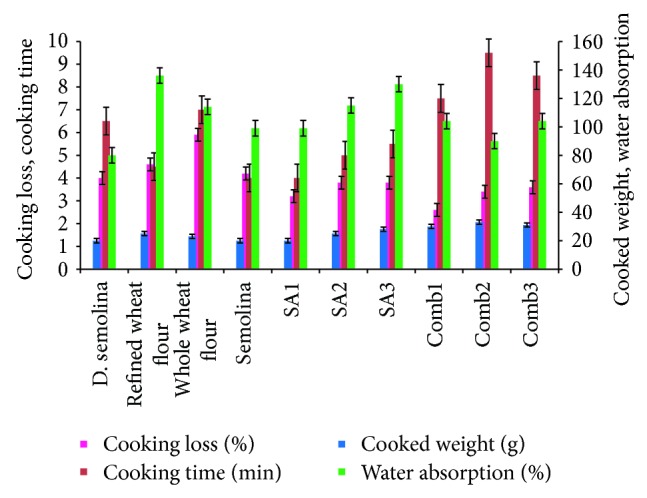
Comparison of cooking quality in various samples. *D. Semolina*: Semolina (*T. durum*). Refined wheat flour: wheat flour (refined). Whole wheat flour-wheat flour (Whole). Semolina: Semolina (*T. aestivum*). SA1: refined wheat flour (50%) & Semolina (50%). SA2: refined wheat flour (50%) & *D. Semolina* (50%). SA3: semolina (50%) & *D. Semolina* (50%). Comb1: *T. aestivum* wheat flour (50%) & semolina (50%) with additives. Comb2: refined wheat flour (50%) & *D. Semolina* (50%) with additives. Comb3: semolina (50%) & *D. semolina* (50%) with additives.

**Figure 4:**
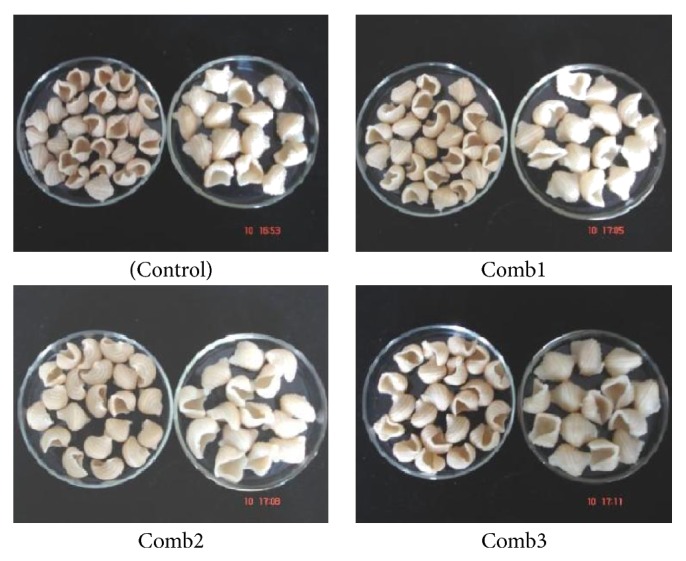
Photographs of raw and cooked pasta samples.

The cooking characteristics of all the raw materials and the trails along with the COMB blends compared to the control are presented in Figure 3 and the photographs of raw, and cooked samples compared to the control are shown in Figure 4. High-quality pasta has a good cooking resistance and firmness, does not release amount of organic matter into the cooking water, and does not show stickiness. The cooking loss of all the Comb samples is much lesser when compared to the control. The lowest cooking loss is seen in (Figure 3) where it is 2.88% compared to the control, which is 4.05%. Cooking loss of ≤8% is considered acceptable for good-quality pasta [11]. Pasta quality and cooking characteristics are dependent upon the protein—starch matrix of the extruded pasta product. The quality of the cooked pasta can be better explained on the basis of the interactions between starch and gluten whose intensity is strongly dependant on the drying conditions, as reported by many authors [22]. If the coagulated gluten structure lacks compactness and elasticity, the starch granules structure swell up easily during cooking and lose more soluble materials into the cooking water.

Starch was the main component (63.1%) on dry basis of the cooking liquor when 100% semolina was used in spaghetti and vermicelli samples. Drying at high temperature (≥60°C) generally results in decreased cooking loss. High-temperature drying strengthens the Gluten matrix, which protects starch granules from rupturing during cooking. Furthermore, high temperature drying reduces water permeability and a crake in packaging & arrangement of starch granules, contributing to reduced cooking looses and increased cooked firmness [23]. A reconstitution study [24] indicates that the gluten quality is the major factor determining the cooking quality. This could be very well noticed in the Comb samples where gluten was added, thus decreasing the cooking loss. Furthermore, pasta should be resistant to overcooking and maintain its shape during swelling. This is also noticed in the Comb samples, where the presence of hydroxypropyl-methylcellulose (HPMC) added as an additive is noted to increase water solubility and also at high temperatures forms a gel creating a temporary network of hydrocolloid chains, thus maintaining the structure [25].

#### 3.3.3. Texture

**Figure 5:**
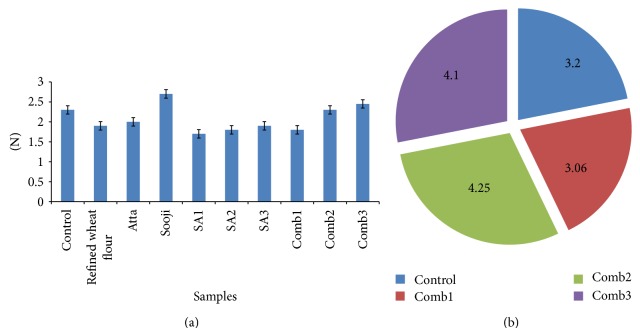
(a) Comparison of firmness in various samples SA1: refined wheat flour (50%) & semolina (50%); SA2: refined wheat flour (50%) & *D. semolina* (50%); SA3: semolina (50%) & *D. semolina* (50%); Comb1: refined wheat flour with additives; Comb2: refined wheat flour (50%) & *D. semolina* (50%) with additives; Comb3: semolina (50%) & *D. Semolina* (50%) with additives. (b) Comparison of Dietary fiber content in pasta. where: Control (*D. semolina*): semolina (*T. durum*). Comb1: *T. aestivum* wheat flour (50%) & semolina (50%) with additives. Comb2: refined wheat flour (50%) & *D. semolina* (50%) with additives. Comb3: semolina (50%) & *D. semolina* (50%) with additives.

The main criteria generally accepted to access the overall quality of the cooked pasta are based on the textural evaluation. The work done by the probes to cut the pasta was directly correlated with the peak force. Cooked firmness was greater for semolina than refined flour or whole wheat flour as seen in Figure 5(a). Whole wheat pasta was reported to have lower cooked firmness than traditional semolina [26]. When cooked to optimum, firmness was seen the greatest in semolina (semolina) obtained from *T. aestivum* wheat but is deemed not suitable for the consumer as it fails at the other parameters. It is also noticed that the addition of additives has resulted in firmness, which is similar to that of control. This can be explained as gluten present is seen to improve the protein content, thus improving firmness of pasta. Cooking quality improves with increased protein content [24]. Good-quality pasta should be al dente; that is, it should have high degrees of firmness and elasticity. Comb3 pasta has moisture values higher than that of control, and at the same time, its firmness showed increased values. This would suggest that the substitution of additives to 100% semolina contributes to structural strength. In this case of Comb3, this hypothesis could also be related to the low values obtained for cooking losses, indicating a well-formed structure from which small amounts of solids are released during cooking.

Peak force is strongly influenced by the cooking time. Increase of the cooking time resulted in a decrease in the peak force. Longer cooking times resulted in an increased water absorption that led to moisture migration into the centre of the pasta. Hence, we hypothesize that moisture migration into the centre of the pasta diminishes the resistance of the sample to cutting, since the plasticizing action of water increases the mobility of biopolymers chains. Another factor that could have affected the texture was the more prolonged exposure to heat while drying that changed the structural conformation of the protein-starch network. This phenomenon may have caused loss of rigidity in the structure with a consequent decrease in firmness.

#### 3.3.4. Sensory Evaluation

A product, even if it is highly nutritious, but does not taste good, will not be accepted in the society, thus making sensory evaluation very important and a crucial criteria in the formulation of pasta. Based on the different properties of importance, it is characterised and formulated as shown in Table 6. It was noticed that the overall score when compared to the control is slightly less compared to all the other samples. Refined wheat flour considered being the alternate for semolina as a raw material is considered to be the second control. The main aim of the present study to find a suitable alternate for the control (durum semolina) thus needs an overall score to at least at par to be acceptable. It is noticed that additives improved the sensory scores especially the mouth feel character. The increase in mouth feels score (Table 6), which is one of the main attributes for pasta sensory evaluation, may also be attributed to the increased water absorption (103.8%, 94%, and 104%), respectively, for the comb blends. Appearance and strand quality has also been increased with the addition of the additives. Comb3 mixture of semolina of *T. aestivum* and *T. durum *gives the highest overall score, which is very much similar to the control.

#### 3.3.5. Dietary Fiber

Being very essential for the bowel movement and the digestion in the body, dietary fiber levels are much noted in the present world. Remarkable changes in the levels of fiber are noticed in the Comb2 and 3 samples, where they are 4.25 and 4.1, respectively (Figure 5(b)). Higher levels of dietary fiber are mostly recommended in the present world, owing to the increase in diet-conscious people and the increased digestive problems faced in today's society. The link between dietary fiber and human health is well explained by [27] where they explain that higher levels of dietary fiber increase cancer prevention, lower risk of chronic disease and help in diabetes management and prevention. It was also noticed by [28] that incorporation of a combination of blends of wheat bran by-products increased the dietary fiber content.

### 3.4. Scanning Electron Microscopy

**Figure 6:**
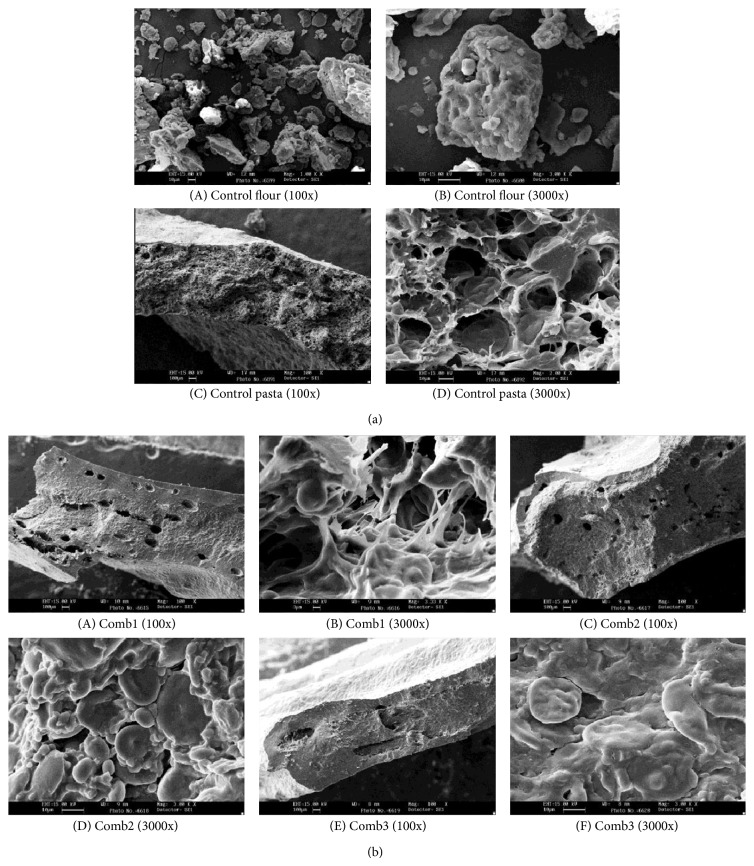
(a) Scanning electron micrographs of control. (b) Scanning electron micrograph of combination blends of pasta, where: control flour: semolina (*T*. *durum*). Control pasta: semolina (*T*. *durum*). Comb1: *T. aestivum* wheat flour (50%) & semolina (50%) with additives. Comb2: refined wheat flour (50%) & *D*. *semolina* (50%) with additives. Comb3: semolina (50%) & *D*. *semolina* (50%) with additives.

SEM techniques were used to investigate the structural integrity of cooked pasta. Two views of SEM showing the cut section and the network of the pasta are presented in Figure 6(b). These micrographs are all presented in 100x and 3000x, respectively, for all of the samples. Micrographs of the control pasta samples show the protein-starch matrix to be well formed, with strong and continuous protein strands entrapping large starch granules. The starch granules within the pasta appear to be slightly swollen and regular in shape and size, perhaps indicating a level of gelatinization during the extrusion process. It is also noticed that the control has smaller pores when compared to the comb blends. This supports the higher cooking loss (4.05%) and lower cooking time (6.5 min) as boiling water can reach the deeper core faster and stay in the cavities. The addition of refined wheat flour to the semolina is seen to disrupt the continuity of the protein matrix. The protein-fiber matrix within the pasta containing a portion of refined wheat flour appears to be less developed than the control resulting in an open appearance with discrete starch granules, which is uncovered and exposed to enzymatic attack. This may be explained by the dilution of the gluten protein, thus showing reduced firmness (1.757 N) and increased water absorption (103.8%), making it a more elastic structure. The addition of semolina with refined wheat flour shows a protein starch matrix similar to that of the control. This sample is seen to have a gelatinized structure when compared to the other blends, thus forming a more compact structure, which enable reduced water absorption (92%) during cooking. Unswollen starch granules can still be found inside the durum wheat, thus reducing the water absorption during cooking due to the layer of gelatinized starch. Use of whole semolina shows a higher network of protein-starch matrix, thus resulting in a higher degree of firmness (2.43 N) and improved chewiness.

### 3.5. *In Vitro* Starch Hydrolysis

**Figure 7:**
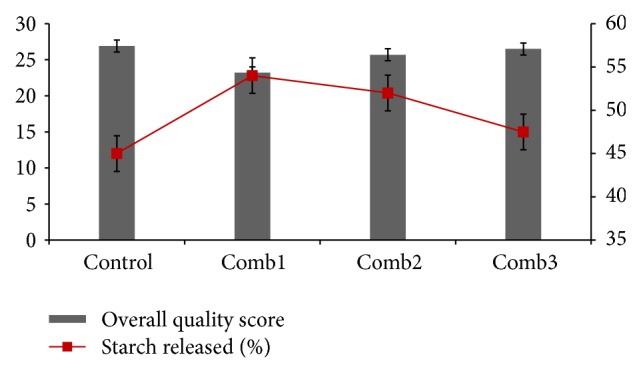
Effects of overall quality score and percentage of starch released in pasta samples. *D. semolina:* semolina (*T. durum*); Refined wheat flour: wheat flour (Refined); whole wheat flour: wheat flour (Whole). Semolina: semolina (*T. aestivum*); SA1: refined wheat flour (50%) & semolina (50%); SA2: refined wheat flour (50%) & *D. semolina* (50%); SA3: semolina (50%) & *D. semolina* (50%). Comb1: refined wheat flour with additives; Comb2: refined wheat flour (50%) & *D. semolina* (50%) with additives. Comb3: semolina (50%) & *D. semolina* (50%) with additives.

The rate of starch digestion and absorption seems to be a determinant of the metabolic response to a meal. The GI is defined as the postprandial incremental glycemic area after a test meal, expressed as the percentage of the corresponding area an equi-carbohydrate portion of a reference food (glucose or white bread). A recent joint FAO/WHO expert consultation recommended increased consumption of low-glycaemic index (GI) foods. There is increasing evidence that a low -glycaemic-index diet can be beneficial in that, it improves metabolic control of hyperlipidaemia in diabetic patients as well as in healthy subjects. The rate and extent of starch digestion instigate a number of physiological functions that have different effects on health, including reduction of the glycemic and insulinaemic responses to a food, hypocholesterolemic effects, and protective effects against colorectal cancer.  Figure 7 shows the total starch of various pasta samples. The values ranged from 44.9 to 53.45%. It is interesting to note that the addition of refined wheat flour as a raw material to the pasta sample increased the starch released in the pasta where pure refined wheat flour as pasta shows the highest level of starch release (44.90%). This is because as refined wheat flour consists of only the endosperm of the wheat grain, thus removing the nutritious bran and germ content (Wikipedia, Refined wheat flour). Goñi et al., [29] have reported a total starch content of 74 ± 2.24 for spaghetti, but the starch content obtained for the control pasta is approximately half of the value said above. This discrepancy may probably be due to the use of raw pasta for our analysis whereas Goñi et al. [29] used the cooked samples of the pasta for their analysis. Methodological differences in determination of total starch may also be a reason for the discrepancy. There are many factors that may influence the rate of starch digestion, including the nature of starch, the starch-protein interaction, the presence of fiber and antinutrients such as lectins, phytates and enzyme inhibitors [30] and method and time of cooking.

## 4. Conclusion

The present study revealed that pasta could be made using different wheat-milled products, which are economically viable and also have beneficial nutritional applications. Different combinations of mixtures had been experimented to produce viable alternates where different concentrations of refined wheat flour, whole-wheat flour, and semolina of different varieties of wheat were used to prepare pasta. Pasta made from refined wheat flour was seen to have reduced cooking loss, colour, firmness and sensory score. Pasta made from a combination of *T. durum* (50%) semolina and *T. aestivum* (50%) has acceptable levels of all the parameters, but due to increase in financial requirements, it is not suitable. From all results obtained, it was seen that semolina *T. durum* (50%) mixed with refined wheat flour *T. aestivum *(50%) can be used as alternate for making pasta for use as a low glycemic index snack product with acceptable physical and sensory properties. The addition of additives to the pasta has made it almost equivalent to the standard of original 100% semolina pasta. Acceptable cooking quality parameters were obtained in the pasta samples containing a mixture of both semolina and refined wheat flour, as measured by cooked weight, cooking loss, and so forth during cooking. Dietary fiber, sensory and firmness also were in the acceptable range. However the incorporation of additives in the mixture of 50% semolina (*T. durum*) along with 50% refined wheat flour (*T. aestivum*) was finally concluded as the optimal alternate, because of its almostequivalent properties to the 100% *T. durum* semolina pasta, thus making it a economically nutritious alternate' resulting as a staple food in developing countries.

## Figures and Tables

**Table 1:** Formulation for pasta processing.

Ingredients	Sample code									
	Con	Sem	WF	WWF	SA1	SA2	SA3	Comb1	Comb2	Comb3
*T. durum* semolina (g)	100	—	—	—	—	50	50	—	50	50
*T. aestivum* wheat flour (g)	—	—	100	—	50	50	—	50	50	—
*T. aestivum* semolina (g)	—	100	—	—	50	—	50	50	—	50
*T. aestivum* whole wheat flour (g)	—	—	—	100	—	—	—	—	—	—
HPMC (g)	—	—	—	—	—	—	—	0.5	0.5	0.5
Vital gluten (g)	—	—	—	—	—	—	—	3.0	3.0	3.0
Ascorbic acid (ppm)	—	—	—	—	—	—	—	100	100	100
Water (mL)	**38**	**32**	**37**	**41**	**34**	**36**	**38**	**39**	**38**	**41**

**Table 2:** Particle size distribution of raw materials and its blends.

Mesh size (micron)	Durum semolina (control)	Wheat flour	Whole wheat flour	Semolina	Comb1	Comb2	Comb3
280	39.85	Nil	Nil	37.86	Nil	20.5	38.9
150	12.9	2.52	43.21	8.15	2.0	6.3	10.1
132	18.75	8.58	24.62	13.6	8.5	22.45	16.1
95	9.35	31.3	19.59	11.25	36.0	11.2	14.35
55	17.1	57.8	12.06	27.6	52.0	38.0	20.0

Control (*D. semolina*): semolina (*T. durum*); refined wheat flour: wheat flour (refined); atta: wheat flour (whole); semolina: semolina (*T. aestivum*); Comb1: *T. aestivum* wheat flour (50%) & semolina (50%) with additives; Comb3: *T. aestivum* Semolina (50%) and *D. semolina* (50%) with additives.

**Table 3:** Raw material composition.

Sample	Moisture (%)	Ash (%)	Protein (%)
Control	9.8 ± 0.05	0.96 ± 0.03	12.8 ± 0.12
Refined wheat flour	10.84 ± 0.07	0.66 ± 0.03	12.44 ± 0.12
Whole wheat flour	10.24 ± 0.03	1.03 ± 0.02	10.42 ± 0.14
Semolina	13.49 ± 0.02	0.49 ± 0.02	10.55 ± 0.11
Comb1	10.87 ± 0.12	0.610 ± 0.04	13.70 ± 0.14
Comb2	9.91 ± 0.68	0.712 ± 0.06	15.40 ± 0.14
Comb3	10.18 ± 0.11	0.596 ± 0.07	11.40 ± 0.12

Control (*D. semolina*): semolina (*T. durum*); refined wheat flour: wheat flour (refined); atta: wheat flour (whole); semolina: semolina (*T. aestivum*); Comb1: *T. aestivum* wheat flour (50%) & semolina (50%) with additives; Comb2: refined wheat flour (50%) and *D. semolina* (50%) with additives; Comb3: semolina (50%) and *D. semolina* (50%) with additives.

**Table 4:** Farinograph results for the flour samples.

Sample	Water absorption (500 BU) [%]	Development time [min]	Stability [min]	Tolerance index (MTI) [BU]	Breakdown [min]
Control	64.5	5.3	5.8	29	11.3
Semolina	60	4.2	4	53	6.5
Refined wheat flour	58.1	6.7	7.3	43	10.2
Whole wheat flour	68.1	3.5	3.2	53	5.6
Comb1	60.2	10.2	13.2	21	16.8
Comb2	63.9	3.8	5.9	27	9.8
Comb3	63.6	4.4	4.9	33	8.1

Control (*T. durum* semolina). Semolina (*T. aestivum* semolina). Refined wheat flour (*T. aestivum*): whole-wheat flour: (*T. aestivum*): Comb1: *T. aestivum* wheat flour (50%) & semolina (50%) with additives. Comb2: refined wheat flour (50%) and *D. semolina* (50%) with additives. Comb3: *T. aestivum* semolina (50%) and *D. semolina* (50%) with additives.

**Table 5:** Colour measurement.

Sample	Raw				Cooked			
	*L*	*a*	*b*	Δ*E*	*L*	*a*	*b*	Δ*E*
Durum semolina	69.13	3.42	21.88	31.30	64.49	1.61	22.78	25.52
Refined wheat flour	76.00	1.605	17.55	24.34	67.52	−1.15	13.85	30.86
Whole wheat flour	63.42	5.09	24.81	37.31	62.22	0.48	15.54	36.51
Semolina	76.74	0.66	18.04	23.23	69.49	−1.17	15.33	29.19
SA1	72.06	1.18	21.28	28.6	68.74	−1.81	10.73	29.23
SA2	67.60	2.93	22.84	33.19	67.71	−0.61	17.62	31.73
SA3	67.8	2.18	20.66	31.26	68.65	−0.14	18.10	30.75
Comb1	66.52	1.7	21.30	34.82	69.06	−1.04	12.83	29.05
Comb2	63.98	2.85	23.80	36.73	66.17	0.22	18.93	33.36
Comb3	67.44	2.2	22.64	33.25	66.73	−0.75	15.56	31.87

*D. semolina*: semolina (*T. durum*); refined wheat flour: wheat flour (refined); whole wheat flour: wheat flour (whole); semolina: semolina (*T. aestivum*); SA1: refined wheat flour (50%) and semolina (50%); SA2: refined wheat flour (50%) and *D. semolina* (50%); SA3: semolina (50%) and *D. semolina* (50%); Comb1: *T. aestivum* wheat flour (50%) & semolina (50%) with additives; Comb2: refined wheat flour (50%) and *D. semolina* (50%) with additives; Comb3: semolina (50%) and *D. semolina* (50%) with additives.

**Table 6:** Sensory evaluation of pasta samples.

Sample	Appearance (10)	Strand Quality (10)	Mouth Feel (10)	Overall Quality (30)
Control	9.32 ± 0.44	8.51 ± 0.68	8.97 ± 0.35	26.8 ± 0.43
Refined wheat flour	6.83 ± 0.45	5.28 ± 0.63	6.14 ± 0.82	18.25 ± 1.56
SA1	7.63 ± 0.65	5.48 ± 0.72	6.74 ± 0.58	19.85 ± 1.48
SA2	6.85 ± 0.71	7.21 ± 0.86	6.49 ± 0.86	20.55 ± 1.81
SA3	6.24 ± 1.12	6.22 ± 0.9	7.74 ± 1.39	20.2 ± 1.04
Comb1	7.92 ± 0.83	7.26 ± 0.24	8.02 ± 0.54	23.2 ± 0.96
Comb2	8.32 ± 0.61	8.93 ± 0.43	8.55 ± 0.3	25.8 ± 0.54
Comb3	9.11 ± 0.85	8.6 ± 0.71	8.89 ± 0.45	26.6 ± 0.89

*D. semolina*: semolina (*T. durum*); refined wheat flour: wheat flour (refined); whole wheat flour: wheat flour (whole); semolina: semolina (*T. aestivum*); SA1: refined wheat flour (50%) and semolina (50%); SA2: refined wheat flour (50%) and *D. semolina* (50%); SA3: semolina (50%) and *D. semolina* (50%); Comb1: *T. aestivum* wheat flour (50%) & semolina (50%) with additives; Comb2: Refined wheat flour (50%) and *D. semolina* (50%) with additives; Comb3: semolina (50%) and *D. semolina* (50%) with additives.
